# Extracts

**Published:** 1882-07

**Authors:** 


### Extracts.

---

Colony Treatment of the Insane.—I went to Fitz-James a few weeks after visiting Gheel. And here I may say at once that I think—though I know there are violent sides taken in the whole matter of the comparative merits of the colony system and the close asylum system—I may say that I believe Fitz-James to be a most splendid example of what may be termed the segregation or separation system of treating the insane, and of the curative effects produced by labor and occupation.

On the day of my visit there were 1,000 patients in the colony—and here the word colony may be appropriately used, for Fitz James consists essentially of two main portions, a central asylum and some immense farms, lying about a mile away. The central asylum corresponds in certain main characteristics to any other close asylum. It is its association with outlying farms that gives to Fitz-James its special distinctive features, and in the use that is made of these farms. The farms themselves consist of about 1,500 acres of the most cultivable and excellent land. An omnibus service goes back and forth between the asylum and the farms, conveying outward the insane who are willing and able to work, and bringing in those who are tired and wish to return. Comfortable lodgings are provided for those who are out at work. And here I may note in passing that no threat more effective in inducing a refractory patient to obedience can be found than telling him that he will be taken back to the central asylum. There are no exceptions to the classes of patients received at Fitz-James. As far as the quality of its patients is concerned, Fitz-James is exactly like the institutions on Blackwell's and Ward's Islands. The comparison that I must soon institute must, therefore, in this instance, be an extremely fair one. The main characteristic of the central asylum was a number of large and roomy court-yards, in which were trees, walls, grass plats, plants and benches. These court-yards, of which there were six, communicated directly with the sleeping apartments. I recall very nearly the numbers who occupied these courts or their connecting wards. In the first there were fifty patients, who were old, feeble, convalescent or desiring to escape; in the second, forty-five on trial before going out to the farm; in the third, fifty epileptics; in the fourth, which was the infirmary, there were also fifty sick; in the fifth, forty-six who were "dirty;" in the sixth, ten who were "excited"—in all 251 out of about 850 men who were at the central asylum. Now this is what astonished me, the remaining 600 were out at work. How enormous this ratio compared with our own similar institutions we shall soon see. Another 150 patients, making up the full thousand, were women, subject to the same classification as above.

I saw no form of restraint anywhere in the whole system, except bars on the second story windows of the part used for a hospital. The reason given for this conspicuous lack of restraint was that nearly all the patients, being at work, were extremely docile and did not require restraint. I never saw patients so quiet, calm and interested in their pursuits as in this asylum.

An interesting feature in the central department is the so-called "trial garden," a large tract of cultivated ground, where the patient about to be trusted on the large farms is first tested to see if it will do to send him out.

I next went to the farms and took a note of the condition of affairs; I regret that time will allow me to present, in the briefest manner, a few only of the most salient points. The farms are two in number, large and adjoining, comprising 1,500 acres, and named respectively Fitz-James and Villers. Fitz-James has three departments: first, for men, both paupers and paying; second, for pauper women; and third, for paying women. Of the men and women who paid, little need be said; the average price was $50 per month; their quarters were pleasing; each, as a rule, had his or her own servant, and wandered about the farms and gardens pretty much at will. But the work and occupations of the pauper men were more interesting. I traversed fertile fields, where the crops were being harvested; haystacks and piles of potatoes and other root vegetables gave evidence of the fertility of the soil, while approved and modern tools showed the practical farmer. The patients worked in gangs of from twenty to thirty men, each with an associate attendant. In all 167 were employed here. In the stables were forty horses, in the sheepfold 1,800 sheep; there were also 200 cows, eighty pigs, and 400 hares. All this I mention, not alone to show that there was room for labor for all, but to point out, also, at the same time, the self-sustaining character of this colony. Near by was a blacksmith shop in which the insane were employed, a mill, and a butchery.

But the most interesting of all, perhaps, was the second department—that devoted to the labors of 150 insane women.

A pretty little stream came down through the trees from the neighboring hills, and over a section of this stream was built the laundry; near by was a dining-hall and dormitory, with 150 plates set along the tables in the lower halls, and as many beds on the second floor. In the laundry is done all the washing of the entire institution. Here were washing machines, drying machines and a steam engine of 10-horse power, whose engineer was a woman attendant, and whose *fireman* was an insane woman. Along the edges of the stream, as it passed through the building, were arranged in double and facing rows, fifty patients engaged in washing clothing; they were happy, industrious, and interested; if they didn't want to work, they were at liberty to return to the central asylum. Other women were busy in the drying-room, and still others were ironing and folding. And to direct and manage these 150 women there were simply one chief directress and eight sane employees.

The second farm, Villers, resembled in most respects the first, except that it was devoted more exclusively to agricultural field labor. Here most of the kitchen vegetables of the institution were raised; nothing is sold, but all is consumed by the colony; 130 patients were employed, with fifteen guardians or head workmen; here there were 600 sheep, 32 oxen and 15 horses. The dormitories were comfortable, and the patients apparently contented.

Each farm has its head agriculturist, who in turn is responsible to Monsieur Labitte, the brother of the medical superintendent.

Here again, then, we find, as at Gheel, an administrative, distinct from a medical, service.

I do not wish to criticise our institutions. I must, however, confess, in closing my remarks, that in studying Gheel and Fitz-James, I have been astonished at the industry of the insane and at the large liberty which may be safely and reasonably granted them. Of the curative power of this industry and liberty no one has any doubt, and I will not here delay to prove the point by an appeal to statistics.

It is true that Gheel is exceptional, but clearly the comparison with Fitz-James is a fair one. Gheel in its entirety is probably an ideal which can never be repeated by any other nation, for the simple reason that there is but one village of Gheel, removed from the world's traffic and turmoil, where the inhabitants, by reason of centuries of inheritance, have learned patience, tact, and fearlessness in their dealings with the insane.

But though the "Gheel idea," *i. e.*, the "family system," consisting of a large number of families who would receive into their midst a thousand or more insane, may not be repeatable, the essence of this idea, *i. e.*, a large and reasonable liberty, healthful and sufficient employment, and accustomed and congenial surroundings, is repeatable, but not certainly in any of our great asylum buildings. Gheel teaches us the possibilities that exist in the treatment of our insane. It shows that the insane will work cheerfully if well managed; that they may be trusted, under proper precautions, with great liberty and not abuse it; and that though in the highest degree intractable in restraint, with liberty they become docile.

In Gheel we find our suggestions. In Fitz-James we find these suggestions carried out with a degree of practical, organized, working perfection, which I believe both in its humane and its utilitarian aspects has yet to be equalled either at home or abroad.—*William J. Morton, M.D., in Journal of Mental and Nervous Disease.*

---

Iodoform: Its Excellencies and Its Defects.—The question of the use of iodoform stands to-day in the foreground of interest, and scarcely any surgeon can avoid making practical proof of it. For the last five months in the Augusta Hospital treatment by iodoform has been tried, and an attempt made to settle the question whether the praises lavished upon it have been really justified.

From the year 1828 to the present, iodoform has required a long time to find its way into general use; at last, through the services of Mosetig, it has succeeded in doing so. Since then a copious literature has developed, which has reached a high pitch of enthusiasm, and has not allowed itself to be led astray by occasional announcements of bad results. In the beginning of the present year earnest notes of warning were sounded by Schede, and since that time, in quick successsion, such results have been announced. Mosetig alone denies all evil consequences. In opposition Kuster feels himself bound to communicate his own experiences. Since the commencement of the iodoform treatment it has become prominent that the drug has striking peculiarities in two directions—(1) antiseptic, and (2) the extraordinary high degree of specific efficacy over tuberculous affections of bones. The antiseptic action is surprising. Wounds, that under Lister's gauze dressing were not absolutely aseptic, were seen to heal up rapidly. In extensive necrosis of the lower end of the femur the speaker had seen large wounds, scraped out and filled with iodoform powder, heal completely with lint dressing in two months, whilst with Listerian dressings long-standing fistulæ generally remained behind. More surprising still is its action in wounds that have already given way to decomposition. In a case of empyema, in which, notwithstanding antiseptic precautions, the discharge became sanious, both fever and odor vanished at once after sprinkling one grm. of iodoform over the wound, and the patient is now almost well.

Those tuberculous affections, also, which are often not to be overcome by Listerian treatment, are now almost with absolute certainty conducted to a favorable termination so long as the bone or joint processes are accessible.

Iodoform treatment of the fungous diseases of joints is somewhat less certain, although even here a definite cure is frequently accomplished by "first intention." The author of the paper cannot ascribe any specific action of iodoform in these cases, but considers that the healing up of them leads back to the high degree of antiseptic power possessed by the drug.

By the aid of these brilliant results iodoform gives rise to a series of disturbances that may be classed as (1) local, and (2) general. To the first belongs the peculiarity of iodoform, that it acts as a foreign body upon the wound. (Gussenbauer.) After amputation of the mamma, five days after the healing of the wound, it re-opened, and a fistula remained. Such fistulæ usually heal up very slowly. A second local disturbance is a peculiar phenomenon that originates under the action of iodoform. It is distinguished by a hard infiltration and a color passing into blue. In the case observed, an abscess resulted after the lapse of some weeks. That iodoform is no protection against erysipelas, is a much more disagreeable peculiarity. Since the introduction of the drug into the Augusta Hospital, erysipelas has become much more frequent, and the writer has rarely seen more serious cases, amongst them one fatal.

More important still are the general disturbances called forth by iodoform: first of all, disturbance of digestion. Some patients complain only of the unpleasant taste that renders disagreeable all the food partaken of; in others, a very persistent diarrhœa makes its appearance after some days, often with some admixture of blood. 2. Iodoform fever. It is almost a rule that patients covered with powdered iodoform exhibit a more or less high temperature, analagous to that experimentally shown by the author to be produced by carbolic acid with evening temperature of 40.5 C. (103.4), but without any actual disturbance of health being dependent on it. Often, however, violent headache is added to the pyrexia and the patients die. 3.

Psychical disturbances both of depression and exaltation. Patients become melancholy, lachrymose; in other cases they become insensible, answer with difficulty when called to aloud, the urine passes from them, and when this condition persists the patients die. Others again, have the most eager longing to get away from the hospital, and strive to escape. In many cases the most exquisite double of delirmium tremens was present. The worst case seen by the author was that of a lady, in whom complete delirium developed and carried off the patient. 4 *Iodoform may be a deadly poison and often is.* The author can increase the statistics of deaths from iodoform by two. The first case is not absolutely free from doubt. It was that of a man whose lower jaw was sawn through, three-fourths of the tongue was then removed, and the stump besprinkled with iodoform powder. On the third day he was very unhappy, uttered wild cries, attempted to get out of the window, from the fourth day to the seventh had serious erysipelas, the patient remained insensible, bronchitis set in and he died. Section: The wound of the tongue absolutely aseptic, high degree of catarrh of the bronchi. According to Aschenbrandt iodoform is intensely irritating to the lungs. The second case is more clear. In a case of ovaritomy the author found a large number of thin walled cysts, from which some exudation found its way into the peritoneal cavity. These showed all the symptoms of old peritonitis; in Douglas's pouch was a quantity of thick fetid exudation. The author sprinkled the whole of the inflamed surface lightly with iodoform to the extent of 30-40 grammes. The abdominal wound was closed. All went surprisingly well for the first few days. From the end of the third day, however, mania set in, and on the seventh collapse, and the patient died. Section: In the abdominal cavity the remains of the iodoform were found, but no trace of sepsis.—*E. Kuster, Berlin. Translated by J. E. Burton in Med. Press and Circular.*

---

ARSENIC IN THE TREATMENT OF CHOREA.—Arsenic has always been a classical remedy for chorea; but I think the credit for using it hypodermically in heroic doses in that malady must be given to M. Gagnon, of the Hotel Dieu, Clermont. The preparation used by M. Gagnon is Fowler's solution, and the injections have been proved to be perfectly innocuous. The dose would be from 15 to 40 drops for an adult, and 15 to 30 for children. One or two observations will be read with interest. One case was that of a young woman in whom the chorea was intense; the muscles of the face contracted in every sense, and in an irregular and rapid manner. The head was turned—now to the right, now to the left—and when the patient was seated the body swayed backwards and forwards, and from side to side. Locomotion irregular, and the voice embarrassed and jerky. All these phenomena are exaggerated when the patient is conscious of being observed. The day following her entry into the hospital an injection of 20 drops of Fowler's solution was given; the next day 30 drops were administered; and on the third day the dose was brought up to 40 drops. On the following day a very sensible amelioration was witnessed: the clonic spasms had become less frequent, and the contortions and grimaces of the face became rarer. On the sixth day the contractions had disappeared, when the patient took rest. On the twelfth day—that is, after twelve injections—the cure was complete, and the patient left the hospital. Another case was that of a little girl, aged 9; the chorea was in her more strongly marked. The symptoms were more pronounced than in the last case. She could not walk without support, and when she was seated the contractions became very violent. Lying down she could not rest one moment tranquil. Fifteen drops of liq. Fowler were injected, and the following day the dose was increased to 25 drops; but shortly afterwards vomiting came on, and the dose the next day was reduced to 15 drops. Forty drops administered on the fourth day, and were well supported, the movements diminishing in intensity. For the four succeeding days the dose was 40 drops each day, and the amelioration was always well maintained. Locomotion is effected with regularity, and the contractions have disappeared. On the ninth day of treatment the vomiting returned, and the dose of liq. Fowler was reduced to 20 drops. Two days afterwards she left the hospital cured. If we consider the different remedies employed in chorea, such as arsenic (internally), salicylate of soda (for according to Germain See chorea is nearly always of a rheumatic nature), bromide of potassium, electricity strongly applied to the vertebral column, and followed by chloroform spray, it must be admitted that none of those means were ever followed by such rapid results as that of liq. Fowler hypodermically. It must not be forgotten to add that in no case was there a relapse. —*Paris Correspondent, in Med. Press and Circular.*

---

SELECTING SPECTACLES.—Dr. Reynolds makes some sensible remarks on this point in the *Medical Herald.* He says: "Few persons, not directly concerned, know how difficult it is to get spectacle lenses properly ground. It is the common belief that all jewelers are opticians, and spectacle-peddlers are experts in optics. An instrument devised by Professor Snellen, of Utrecht, enables one to determine with almost perfect exactness, the powers and quality of refracting lenses. This instrument, the phakometer, shows that nearly all the glasses sold in frames ready-made, are but poor imitations of the real thing for which they are sold.

"Crown glass, and not pebble, is the only proper material for spectacle lenses. The only lenses fit for use are those made to order by some fixed system of grading the refracting power.

"It is often observed that the common lenses hawked about the country as *diamond*, *crystal*, etc., are the poorest quality of glass, so imperfectly ground as to represent sectors having different parts of the lens. It is quite rare to find in these an equal refracting power in the two glasses in the same frame. Persons who need glasses to correct presbyopia, are not likely to require anything like accuracy, unless they are given to literary habits. Persons who read much soon find that the ordinary glasses of commerce, and those peddled by the '*professors of optics*,' are not adequate, and seek the aid of a practitioner of ophathalmic medicine who is able to distinguish between muscular asthenopia and neuralgia."—*Medical Record.*

---

Grape Sugar and Glucose.—The terms grape sugar and glucose are used to designate the two forms in which the article is sold by the manufacturer. Grape sugar is the solid and glucose the liquid form. It is made from starch by the action of sulphuric acid, or oil of vitriol. The first stage in the manufacture is the production of starch. This is made from the Indian corn of commerce; that which posesses the largest amount of starch having the preference over corn of smaller kernels and consequently less starch.

The corn separated from the cob is placed in large vats with boiling water, and there undergoes a steeping process of about 48 hours duration, during which time the water is changed occasionally; this serves to swell the kernel to nearly twice its natural size and softens it very materially.

The mass is then run through mill-stones, which grind it to a fine pulp. With a large percentage of water in this, there are the starch, gluten and woody fibre, etc. In order to separate the starch from this, the pulp is run over large frames covered with bolting cloth, similar to that used in flour mills; several small streams of water are also introduced, and water falling on the pulp as it passes over the sieve, separates the starch from the hull which passes off, the starch falling into a trough underneath, from whence it is conveyed to tubs of larger size, where it is allowed to settle, and the water, which has, by this time, accumulated large quantities of dirt, etc., is allowed to drain off. Caustic soda is then added in about the proportion of 600 lbs. to each 1000 bushels of corn; this serves to further separate the starch from the gluten. From the settling tubs it is allowed to run through long inclined troughs of a very slight elevation. While the water, containing large quantities of starch, gluten, etc., is passing over these tables, as they are called, the starch slowly settles to the bottom, allowing the dirt, gluten and alkali to pass away.

The now solid mass of starch is taken away from the tables and after being broken up thoroughly in water, is pumped into tubs and agitated thoroughly; this further cleans it. The result of this last manipulation is a clean starch, such as is used on our tables in pudding, etc., and in our laundries to stiffen and improve our shirts and other articles. The starch mixed with water is then poured into a large tub called a *converter*, and it is here that the starch loses its identity, and in a high temperature is converted by the action of sulphuric acid into either grape sugar or glucose.

If glucose is desired, the liquid is boiled until, by the application of the iodine test (known to chemists as the method for determining the existence of starch), it shows that it has very nearly lost its character as starch, and the conversion is then stopped by drawing off the liquid. Should grape sugar be desired, the process is carried still further until not a trace of starch is visible, and the liquid is all converted into sugar. We will now follow it from the converting tub to another large tank in which it lies perfectly passive, but possessing in its muddy looking mass a powerful agent and the one source from which are derived the many ideas regarding its poisonous character. I refer to the sulphuric acid. Were the substance left as it now is, and the article put upon the market, it would certainly serve to be severely handled by physicians, parents, and all interested in the health of the people at large. It is a well-known fact that if marble dust is introduced into a liquid containing sulphuric acid, a chemical change takes place, and if a sufficient quantity is introduced, the acid in its entirety is absorbed, and the two together become sulphate of lime, a precipitate easily removed. This is the course pursued in neutralizing the acid and to this end a test paper is applied to determine when the neutralization has taken place; when this is accomplished the liquid is a sweet water, quite dirty, however; it is then passed through filter cloths of various degrees of fineness, and they eliminate a portion of the dirt, including the sulphate of lime. This water is then allowed to run through a larger tank containing burnt bone or charcoal. This takes unto itself what dirt has escaped the former filtering medium, and the now clear liquid, although nearly as thin as water, is quite sweet. It is then introduced into a copper pan of large capacity, called a vacuum pan, and the process of evaporation goes on. As the liquid is slowly evaporated under the influence of heat, it assumes a thick, gummy character of a density of 38° to 42° Beaume. It is now ready for cooling and shipping. Should it be grape sugar it is drawn into barrels or boxes, where it rapidly cools and becomes solid and perfectly white; should it be glucose, and intended for confectioners' use, it is drawn into barrels, from which, owing to its density, it is quite difficult to abstract it unless by heating. Should the manufacturer wish to fill an order calling for syrup, he mixes with the still warm glucose about 10 per cent. of its bulk of cane syrup, a liquid obtained from cane sugar during the refining process. This syrup serves to sweeten the not too sweet glucose, and imparts to it a bright yellowish color called by grocerymen "golden," and so labelled on their syrup cask. The grape sugar maker sells to outside parties the sugar in solid form. They, by the use of patented machinery, cut it into minute pieces and mix it with the coffee sugars of commerce, in the proportion of 80 per cent. cane sugar and 20 per cent. grape sugar. This proportion will vary somewhat, but is in the main correct. The appearance of the mixture is a very decided improvement over the appearance of the pure cane sugar, due to the fact that the cane sugar, being of a yellowish color, is made to look much lighter, when mixed with the white grape. The consumer, unless very well posted, cannot detect the difference, and obtains sugar of much lighter color for the same amount of money that he would otherwise pay for a dark, yellow grade.

The confectionery trade uses a large quantity of both glucose and sugar in the manufacture of all grades of confectionery. Brewers are very large consumers, as well as jelly and wine manufacturers; printers mix glucose with glue and use it for inking their type. Thus it is rapidly becoming a popular necessity. It cheapens the food and when properly made does not hurt the stomach. There is only the one chance for it to be injurious to the human system, and that is in the existence of acid, which cannot occur if the manufacturer is careful to thoroughly neutralize it; if this is done it contains nothing that is harmful. It is estimated that the entire consumption of corn the ensuing year, for this purpose, will be between ten and eleven millions of bushels, of which quantity Buffalo alone requires about six millions.—*J. M. Tallman, in the Physicians and Surgeons Investigator.*

---

Ulcer of the Rectum—In some cases you will find that the lesion is extremely minute, probably no larger than a fissure such as one often has upon the lip in cold weather. Again, there may be an ulcer as large as your thumb nail. In the course of time the ulcerated surface becomes hypertrophied, its edges raised or the centre depressed; in fact, you may find a variety of pathological appearances connected with these lesions; but there is one complaint that will always exist, namely, hyperæsthesia; a hyperæsthetic condition of the nerves distributed to the base of the ulcer.

Formerly this condition of things—the pain excited by these sensitive nerve filaments—was treated by dividing the muscular fibres, the sphincters, whose contraction kept up the compression and motion in the parts; but latterly it has been treated by stretching the muscles to such an extent as to produce a temporary paralysis. In order to perform this operation the patient must be under the influence of ether, and, indeed, it often happens that one is unable to make a satisfactory examination of the patient's condition until she is placed under the influence of an anæsthetic.

The pain inflicted by stretching the sphincter is entirely too great to admit for a moment of our doing the operation without an anæsthetic, although before this agent was widely employed the treatment was sometimes resorted to. The surgeon gradually introduced all his fingers up into the rectum, and then doubled up his fist and suddenly, in that position, jerked it through the sphincter. You can imagine the sufferings of the poor creature who might have happened to be the patient.

After oiling my fingers, they are gradually introduced into the rectum, and outward traction force is made in either direction, with the hands, gradually increasing it so as to stretch and not rupture the fibres of the sphincter. If sudden and too great force is used the muscles will be lacerated to a greater or less degree, and there will be incontinence of the fæces, for we seldom find rupture of this muscle tending to repair thoroughly. Unquestionably, even as carefully as I have stretched the parts, certain individual muscle fibres have been ruptured, but they have been ruptured, not in masses, but at different points of the circle, and the damage done to the muscle as a whole is very soon repaired, and after healing it will nearly have regained its normal power. Four or five minutes may be employed in the stretching process, passing the finger around from one side to the other of the muscle, so as to have the distention equal in all directions.

Having stretched the muscle sufficiently, there is no speculum which will enable you to examine the parts better than it can be done with Sims', aided by an ordinary depressor. Upon the posterior wall I find the location of the difficulty, which consists in an ulcerated surface about as big as the nail of my ring finger. It is superficial, involving the mucous structure; in its centre is a small depression. This will require some other treatment in addition to the stretching of the sphincter which has been done, and there is none better than applying to it fuming nitric acid, washing the surface then with the bicarbonate of soda, so as to prevent the too deep action of the remedy.

It is not unlikely that a second application of the acid will have to be made before this ulcer will heal. Such would not be the case, probably, if there were present simply a fissure. Ulcers of the rectum are among the most difficult we have to treat, and especially is this the case if there be also some constitutional disturbance, as syphilis, in which case the lesion is possibly due to the deposit of syphilitic material. In addition to the local treatment there should be constitutional or general treatment.

With regard to general treatment, the first point regards the bowels. These patients always require such medicines as produce soft movements. In addition to this you must see that the general health is cared for. If there be failure of nutrition, besides proper diet, no remedy will be of more service than cod-liver oil. Arsenic, quinine, strychnine, and iron may be given according as they may be indicated. With regard to her uterine condition, that will be treated on a subsequent occasion.—*William M. Polk, M.D., in Med. and Surg. Reporter.*

---

Sewer-Gas.—This term has been used to designate the gas formed by the decomposition of animal and vegetable matter, whether in the sewer, cesspool, or sink. According to the analysis of Dr. Letheby, of London, it consists of sulphuretted hydrogen, sulphide of ammonium, carburetted hydrogen, oxygen, nitrogen, and carbonic acid gas, and organic matter. Fortunately the first-named constituent is usually present in sufficient quantity to make its presence manifest by its odor, and sometimes it is able to blacken paper that has been impregnated with nitrate of lead. This test-paper is not, however, a safe test for sewer gas, as the more poisonous constituents are without action on lead paper. Sulphuretted hydrogen is stated, in all the text books, to be exceedingly poisonous. This is no doubt true of the *undiluted* gas, but chemists who are compelled to work in an atmosphere strongly impregnated with sulphuretted hydrogen for a long time suffer no injury from its inhalation mixed with atmospheric air. Bunsen also says that there is no danger from this gas when mixed with air. Then, too, it is very easily decomposed by oxidizing agents, like chlorine, or absorbed by metallic salts, such as chloride of lead. Its odor is such that none but the most careless will neglect to ventilate a room in which they find any considerable quantity of this gas. Having shown that sulphuretted hydrogen is one of the least dangerous constituents of sewer gas, we pass by the other gases to speak of the organic matter, that subtle poison which no reagent can detect, no microscope discover. It is now pretty generally known that in the decomposition of animal and vegetable matter, but especially of fæcal matter, the most dangerous products are evolved. Whether these are gases or minute organisms, we cannot positively assert, but no doubt remains of their disease-producing power.

Sewer malaria, as it has been called, does not kill people outright, but it is the source of many diseases which themselves destroy life, and among the acute diseases which it will generate is typho-malarial fever. The congestion of any organ may arise from the breathing of so-called sewer gas. It robs man of his strength and woman of her beauty; it debilitates the system and hurries its victim into a premature grave.

In addition to the danger incurred from typho malaria and other diseases engendered by ordinary sewer gas, there is still another danger, not to be forgotten, namely, the means which sewers afford for the spread of contagious diseases. The germs, or poison, of scarlet fever appear time and again where the disease is not expected. Is it not probable that they are carried down the drains of houses to the sewers, in water which has bathed the bodies of the sick, or washed their clothes, and are conveyed to a distant part of the city, to be again forced back by the gas, which is generated in the sewers, into other houses? No doubt many a contagious disease is spread over a city in just that way.

The ordinary disinfectants are of little use against sewer gas. The only thorough and efficient disinfectant is *fire.* Pass the gases through flame and they are rendered harmless. Some disinfectants, like chloride of lime and carbolic acid, have so strong an odor of their own that they are able to mask the smell of sewer-gas, and the deluded householder believes he has destroyed the latter gas. Others, which are odorless, like the salts of zinc, iron and lead, absorb the sulphuretted hydrogen, that most useful indicator of sewer-gas, and render it doubly dangerous. Professor Chandler compares this to pulling down a red flag on a house that contains a case of small-pox. Danger is always increased by removing the danger-signal, and disinfectants too often give a feeling of security which is false. The very bad odor alone of what is called water-gas renders it safe to use it in our dwellings; even the common illuminating-gas would be too dangerous for ordinary use if it had no smell. Coal-gas, the poisonous constituent of which is carbonic oxide, has a very perceptible odor, which often protects us from its evil influence by giving timely warning of its presence.

Disinfection is useful, especially in contagious and other diseases, but cannot be relied upon to rob sewer-gas of its danger. "The use of these modern abominations, which pretend to kill the germs of disease, patent disinfecting machines, water-closet purifiers, and so forth, is pernicious." Health Commissioner DeWolf says: "Disinfection is necessary at all times, but the disinfectant most easily obtained and applied is fresh air. With this best of all remedial and hygienic agencies, I would keep all houses and rooms constantly supplied. The germs of disease are floated hither and thither, and fed by an impure atmosphere. In pure air they are destroyed, or, at least, lie dormant."

Having briefly sketched the dangers of sewer-gas, and shown that it is useless to fight it with disinfectants, we conclude that our only safety consists in driving it out of our houses and shutting it out.—*Boston Journal of Chemistry.*

---

HINTS FOR THE DIAGNOSIS OF OVARIAN TUMORS.—Dr. A. Macdonald gives the following hints in the Edinburgh *Medical Journal:*

1. *Pregnancy*—The possibility of pregnancy, the signs and symptoms of pregnancy, and waiting if in doubt, place the diagnosis beyond possible mistake, with a fair measure of care.

2. *Fibroid.*—A large fibroid with solid walls, leading to general enlargement of the uterus, is easily diagnosed. The increased length which the sound enters, and the fact that the uterus moves with the sound, and the peculiar feel of the uterus, and the nearly constant menorrhagia, suffice to keep the diagnosis correct. It is quite common to hear a bruit in a case of uterine fibroid; only in vascular sarcomata is such audible if the tumor is ovarian.

But much greater difficulty is experienced in cases of fibro-cystic tumors connected to the uterus, with or without pedicle. In that case we must try to ascertain whether the tumor is connected or disconnected with the uterus. Then the cyst of a fibro-cystic tumor may be tapped, when we expect to find only a thin fluid of great density, with some blood-corpuscles, and possibly some non-striped muscular fibres. But in those cases it is often found that only an exploratory incision can determine the diagnosis with accuracy.

3. *Renal Cysts* begin below the false ribs and extend downward and forward. They have a line of resonance between them and the liver, due to the transverse colon; which is of value, as showing they are not of hepatic origin, and when aspirated contain urea. Usually accompanying such there are urinary symptoms, but not always.

4. *Ascites* exhibits the characters of free motion of fluid in an imperfectly filled cavity. Accordingly, when the patient lies on her back the abdomen is flattened anteriorly, the flanks give a dull note, and there is clearness round and above the umbilicus. With change of the patient's position the areas of resonance alter. Thus if the patient is turned on her left side, the right flank gives a clear note, and *vice versa.* In case of tapping, as ascites, the thick gelatinous fluid characteristic of ovarian tumor is never obtained.

5. *Hydatid Cyst of the Liver.*—In this case the tumor grows from the liver, distending first the distance between the ensiform cartilage and the umbilicus, the reverse of an ovarian cyst. Again, tapping and discovering acephalocysts in the fluid is convincing evidence of the true nature of the tumor.

6. *Hysterical Abdominal Distension*, commonly known as spurious pregnancy, need deceive no one, as the percussion is uniformly resonant, and the tumor disappears under chloroform.—*Canada Med. Record.*

---

Questionable Surgery—Oophorectomy.—The operation introduced by Dr. Battey is, unfortunately, being widely performed in this country. It is perfectly safe to assert that on no organ of the body are more doubtful operations performed than on the uterus and its appendages, and that in no department of medicine is the intellectual crippling of specialism more signally demonstrated than in that of obstetrics. Greed and the predilection engendered by special and limited study are apt to compel men to unravel all forms of disease from the standpoint of the particular department of which they may happen to have taken parental charge. This is daily illustrated in the experience of every practitioner who chooses to have his eyes and his mind open to conviction. Quite recently, at the Obstetrical Society of London, Dr. Braithwaite, of Leeds, read a paper "On Two Cases of Unilateral Oophorectomy," the first of which was performed for a *cardiac affection* associated with dyspnœa! and the other for pain in the left ovarian region. We thoroughly endorse the the remarks of the President—Dr. Matthews Duncan—on these cases, that "to remove one ovary as a treatment of cardiac dyspnœa he regarded as a wild proceeding; nor could he imagine that it ever could come within the range of rational medicine." Surely the unfortunate women thus operated on do not properly apprehend the nature of the operation to which they are subjected. It is not so long ago since obloquy and contumely were showered on an unquestionably able surgeon—the late Mr. Baker Brown—for the operation of clitoridectomy. We seem to have made rapid strides since then; yet, we have no hesitation in saying that in the cases indicated by Baker Brown, clitoridectomy was an infinitely more justifiable operation than oophorectomy. We hope to hear less of this barbarous operation in future. At the same time we would protest against the indiscriminate examination of women at public institutions before crowds of students as demoralizing to all concerned. At a certain *clinique* for women, in Scotland, we understand that the vast majority of women who present themselves are examined with the speculum in the presence of the students, and the *os* daubed with "iodised thymol" for all conceivable diseases. This disgusting and degrading practice should be circumscribed not less in public than in private. It is saddening to reflect on the amonnt of mischief which is fairly chargeable to meddlesome surgery in the course of one single year.—*Med. Press and Circular*, (*London.*)

Apropos of this criticism the following letter is reproduced:

BIRMINGHAM, ENGLAND, May 1, 1882.

*Editor of the Chicago Medical Review:*

SIR:—My attention has just been called to a paragraph in the *Chicago Medical Review*, in which you contrast the frequency with which Dr. Battey and I perform this operation. The explanation is simple, and given in Dr. Battey's own words to me is to the effect that if he could get the same results as I do in such operations, he would do them as often. What the explanation of this difference is I do not pretend to say, but my mortality stands at little more than three per cent. Yours truly,

LAWSON TAIT.

The sympathy of the profession under these circumstances must necessarily be with Dr. Battey.—EDITOR OF REVIEW.

---

DOCTORS IN NORWAY.—Not far from the parsonage, high up and overlooking the lovely landscapes of the fjord, was the new home of the doctor who had met me at Skibotten, and I had come to make the promised visit. He had been appointed doctor of the district but a short time before, and had just bought the farm. When I arrived he was not at home, but his young and amiable wife received me with great kindness, and bade me await her husband's return; she was expecting him every minute; he had gone to see some patient. While waiting for him, his wife having gone to prepare a meal, I looked around. The house was a picture of neatness; there was evidence of refinement everywhere in this plain unpretending home —books, music, engravings. In a short time the doctor came. He was cold and tired, for the sail had been a long one, as the patient lived far away. The district to which he had been appointed by the government was a large one. Throughout Scandanavia there are *distrikts læge* (doctors of a district), who received yearly a certain amount of remuneration from the state, varying according to places; for the population is so scattered that, were it not so, large tracts of country would be found without medical help, for doctors could not make a living. The fees they receive are regulated by law, according to the distance traveled from their residence. Hard, indeed, is the life of country doctors, especially of those whose districts are on the fjords or by the sea coast. The only way of locomotion they have is by boat. Often they have to sail or row some twenty or thirty miles, or even more, and encounter all sorts of weather; and great praise is due them for their lives of self-denial. I did not wonder they were so highly esteemed by the people.—*DuChaillu's Land of the Midnight Sun.*

---

Eggs as Food.—Eggs, at average prices, are among the cheapest and most nutritious articles of diet. Like milk, an egg is a complete food in itself, containing everything necessary for the development of a perfect animal, as is manifest from the fact that a chick is formed from it. It seems a mystery how muscles, bones, feathers, and everything that a chicken requires for its perfect development are made from the yolk and white of an egg; but such is the fact, and it shows how complete a food an egg is. It is also easily digested, if not damaged in cooking. Indeed, there is no more concentrated and nourishing food than eggs. The albumen, oil and saline matter, are, as in milk, in the right proportion for sustaining animal life. Two or three boiled eggs, with the addition of a slice or two of toast, will make a breakfast sufficient for a man, and good enough for a king.

According to Dr. Edward Smith, in his treatise on "Food," an egg weighing an ounce and three-quarters contains 120 grains of carbon, and seventeen and three-quarter grains of nitrogen, or 15.25 per cent. of carbon and two per cent. of nitrogen. The value of one pound of eggs, as food for sustaining the active forces of the body, is to the value of one pound of lean beef as 1584 to 900. As a flesh-producer, one pound of eggs is about equal to one pound of beef.

A hen may be calculated to consume one bushel of corn yearly, and to lay ten dozen or fifteen pounds of eggs. This is equivalent to saying that three and one-tenth pounds of corn will produce, when fed to a hen, five-sixths of a pound of eggs; but five-sixths of a pound of pork requires about five pounds of corn for its production. Taking into account the nutriment in each, and the comparative prices of the two on an average, the pork is about three times as costly a food as the eggs, while it is certainly less healthful.—*Boston Journal of Chemistry.*

---

Habit.—When a living being performs an act under the operation of certain impressions which are received, there is a tendency toward the performance of a similar act if like influences are brought to bear upon the organism.

This disposition to repetition is not limited to physical acts; it prevails in regard to almost every function of the body and mind, and forms often an important element in the promotion of disease.

Habit, therefore, is periodicity, and may be defined as the disposition which the organism acquires from the frequent performance of certain acts to repeat these acts till some more powerful force intervenes.

Again, it is well known that the impressions, or consequences which result from the action of certain agents, become less marked if the operation of the cause be repeated. Thus the system becomes habituated to the action of alcohol, opium, chloral, and many other substances, so that while a small quantity will, in the first instance produce the characteristic result, the dose must gradually be made larger or be more frequently repeated in order to be followed by a corresponding effect.

The influence of habit over the ordinary operations of the economy is plainly seen. The sensations of hunger and thirst are experienced at stated periods of the day, because by frequently eating or drinking at those times the system, as it were, expects a repetition, and hence the appropriate sensation is experienced. The votary of opium or chloral is subject to the same law, and invariably feels the want of the customary sedative if its ingestion be delayed for even a short time.

The oft repeated impression has left its traces each time, until at last it assumes a local habitation and becomes permanently fixed in the brain, not to be lost unless through some more powerful influence acting in a similar manner to the first.

Besides this, the various organs of the body also become habituatəd to the effects of stimulants ond sedatives, such as alcohol, tobacco, opium, chloral, and the like, and are the seat of various painful sensations if the supply be cut off.

Relative to the propriety of stopping habits which are injurious to the body or mind, there can be no doubt. To do so, often involves the causation of much temporary suffering, but the object to be ultimately gained is of far greater importance, and is well worth all the pain and anguish that may have to be endured.—*Wm. A. Hammond, M.D., in Am. Jour. of Stimulants and Narcotics.*

---

USES OF OIL OF PEPPERMINT.—I have found the oleum menthæ pip. more effective than any other form of anodyne application I have tried, in allaying the painful neuralgic pain, so often piteously complained of in cases of herpes zoster. These distressing pains—worse in elderly people—are complained of often when the eruption has disappeared: but painting the affected parts over with ol. menthæ pip. nearly always affords speedy relief.

I have painted the oil over the eruption when it was out in a fresh florid condition, and that with great relief to the patient.

The value of this application in pains of neuralgic character deserve to be better known than it is.

The oil is also a good test for sewer gas; that is, for finding out if there is any, and if so, where it comes from. It answers better than either creosote or ether according to my experience in this sort of work.

My plan is to pour an ounce, or less, down a water closet or suspected drain with some hot water after it. In this way I have made rather startling discoveries in the way of bad workmanship, faulty soil pipes, etc. It is hardly necessary to say that he who has to smell for the oil along the course of the drain must not have been handling it beforehand, or have been in any way connected with it.—*John Meredith, M.D., in Birmingham Med. Review.*

---

MEDICAL HIGHWAYS AND BY-WAYS.—No matter what theory prevails, or has prevailed, physical, metaphysical, humorist or solidist or animist, there always are and always have been those who are content to study disease as they see it, without regard to any *a priori* opinions or deductive generalization. So there is one broad division running through medical history, sometimes, as in the school of Alexandria, separating medical practictioners into hostile camps; that of the Dogmatics and that of the Empirics—the believers in "*It ought to be*, because my theory says so," and the believers in "*It is*, because *I see it*."

The new birth of anatomy dates from the great work of Vesalius, first published in different editions about the middle of the sixteenth century.

Another century passed before the art of healing found a new teacher in the person of a physician who was born with that power of observation, that tact and sagacity, which are the supreme gifts of the practitioner. In the words of the illustrious Dr. Thomas Young, "Physic is one of those departments in which there is frequent necessity for the exercise of an incommunicable faculty of judgment, and a sagacity which may be called transcendental, as extending beyond the simple combination of all that can be taught by precept." This "incommunicable faculty" is the same quality which Cabanis speaks of as that tact which alone can render available at the bedside all the stores of knowledge and all the powers of reason.

The Father of commom sense practice in England was Thomas Sydenham, "the restorer of true physic," as Hume calls him, a man whose name is coupled with exalted praise in the pages of Johnson and Macaulay, and gives its title to one of the most useful and distinguished British Medical Societies.

You may be surprised that I have not devoted some considerable space to the wonderful discovery of Harvey. But it has been said, and I think with some truth, that no great change in medical practice was effected by this physiological discovery. I will not attempt to investigate the indirect influence it may have had on medicine, and especially on surgery. It is a part of that connected series of ascertained data of the laws of life which, when it does not lead directly to practical consequences, often points the way which experiment is to follow, and on which it remains for experience to pass its judgment.

John Hunter, unscholarly, obscure in expression, uncouth in diction, bears for all that the greatest name since Harvey's in the annals of English anatomical and physiological science.

We find our ourselves brought down to the close of the eighteenth century and the beginning of that which is now in its last quarter.

I need only indicate the leading lines along which the steps of medical progress have passed within the range of our more immediate vision.

The great discovery of the protective power of vaccination against small-pox, made at the close of the last century, came into general recognition in the first years of the present one.

Next to this, among the grand discoveries which irradiate not merely the foot-path of Medical Science and Art, but the great highway of humanity, is that of induced anæsthesia.

Next in importance to the practical application of anæsthesia we may mention the introduction of physical methods of exploration—auscultation, formally brought before the world by Laennec in 1819, and percussion, revived by him after more than half a century of comparative neglect. Of this I can speak with some confidence, for I was once well acquainted with it practically, and I have made many an ante-mortem autopsy with my stethoscope and pleximeter. There is no need of insisting on the value of a method of ascertaining the existence of disease so obviously of great assistance. But it may be carried too far, and I am afraid sometimes is so. I have often felt, when seeing hospital patients worried by hammering and long listening to their breathing, in order that the physician might map out nicely the diseased territory, the boundries of which he could not alter, as if it was too much like the indulgence of an idle, and worse than idle curiosity. A confessor may ask too many questions—it may be feared that he has sometimes suggested to innocent young creatures what they never would have thought of otherwise. I even doubt whether it is always worth while to auscult and percuss a suspected patient. Nature is not, unkind in concealing the fact of organic disease for a certain time. What is the great secret of the success of every form of quackery? *Hope kept alive.* What is the too frequet fatal gift of science? *A prognosis of despair.* "Do not probe the wound too curiously," said Samuel Sharp, the famous surgeon of the last century. I believe a wise man sometimes carefully worries out the precise organic condition of a patient's chest when a *very* wise man would let it alone, and treat the constitutional symptoms. The well-being of a patient may be endangered by the pedantic fooleries of a specialist.

Perhaps the medical use of the thermometer is the next in importance of the medical improvements of our century. But of this I have nothing to tell you which you do not know or will not soon learn. Of the ophthalmoscope and laryngoscope I can say nothing of my own knowledge.

You must permit me to enumerate in the briefest possible manner some others of the changes that mark the progress of the time over which my own knowledge extends, and which belong to the history of medical science.

The treatment by hypodermic injections has enabled the physician to stay the anguish of dyspnœa and arrest the thrills of neuralgia, as the harper stills the quivering cords of his instrument by laying his hand against them. Old measures of treatment have fallen into disuse. Bleeding is almost a lost art, so rarely is it employed.—*O. W. Holmes, M.D., in Boston Med. Journal.*

